# Bioactivities of *Allium longicuspis* Regel against anthracnose of mango caused by *Colletotrichum gloeosporioides* (Penz.)

**DOI:** 10.1038/s41598-020-68399-z

**Published:** 2020-07-09

**Authors:** Dionisio de Guzman Alvindia, Mark Anthony Angeles Mangoba

**Affiliations:** 1 Food Protection Division, Philippine Center for Postharvest Development and Mechanization, Department of Agriculture, Muñoz, Nueva Ecija Philippines; 2 Department of Bio-Resource and Food Science, College of Life and Environmental Sciences, Konkuk University, Seoul, South Korea; 3 Center for Natural Sciences and Environmental Research (CENSER), De La Salle University, Taft Ave., Manila, Philippines

**Keywords:** Microbiology, Plant sciences, Chemistry

## Abstract

The present study focused on the effect of *Allium longicuspis* extracts (ALE) against anthracnose of mango fruit. In vitro tests (mycelial growth and conidial germination) showed that, ALE concentrated from 0.75 to 2.5 g L^−1^ completely inhibited the growth of *Colletotrichum gloesporioides*. Cytoplasmic discharge, mycelial and conidial blasts were clearly observed when applied with ALE. The minimum effective concentration (MEC) of ALE at 0.75 g L^1^ can be applied as protective, curative and simultaneous treatment in mango fruit to inhibit the anthracnose infection. Efficacy of garlic extract was relatively superior to synthetic fungicide based on protective, curative and simultaneous treatments. Twenty chemical components were detected in ALE based on GCMS analysis (Table 1). The six major components were the following: oleyl alcohol, methyl ether (42.04%), γ-sitosterol (15.85%), , 24-norursa-3.12-diene (5.62%), 1-octadecanol methyl ether (4.23%), *n*-pentadecanol (3.95%) and 2-vinyl-4h-1 3-dithiine (3.76%). The findings support the potential use of ALE as an alternative to synthetic fungicide.

## Introduction

*Mangifera indica* L. popularly known as mango, is the second most important agricultural fruit in the Philippines with a production yield of 899.014 metric tons in 2016^1^. Losses is as high as 2–33% due to anthracnose disease^2^. *Colletotrichum gloeosporioides* (Penz.) Penz. and Sacc is a plant pathogenic fungi that dominantly attack mango fruit and main cause of anthracnose disease^3–7^. The fungus is ubiquitous and responsible for many fruit diseases of other tropical fruit such as banana, avocado and many others^6–7^. Furthermore, it infects the inflorescence, young leaves and branches, and its fruit^3,5^.

In the past, synthetic fungicides (dithiocarbamate, benomyl, thiabendazole, prochloraz, imazalil and copper fungicides) are used to control anthracnose infection^3,8,9^. However, some of them are no longer used for mango export in other countries and is somehow restricted because of public concern over exceeding maximum residue limit (MRL)^3^. Further, due to continuous used of synthetic fungicides, fungal pathogen developed resistance and it contaminates our environments^10^. Therefore, the screening of potential fungicides derived from natural products (plants) may help to overcome this problem.

*Allium *spp. on the other hand, has been widely utilized not only as spices for food but also for treatment against wide range of microorganisms including fungi^11–17^. Organosulfur compounds are mainly produced by *Allium* spp. This compound penetrates the cell membrane and undergo thiol-disulphide exchange reactions in proteins^18^. This information is mainly the basis of fungal death^19–20^. Hence, organosulfur compound has multiple targets inside the cell and this fact can make it tough for the pathogen to develop resistance^18^.

Limited knowledge exists on the effect of *Allium longicuspis* or commonly known as “garlic: wild type” against *C. gloeosporioides *in vitro and in vivo tests. This may be useful in managing anthracnose incidence in mango fruit and as an alternative to synthetic fungicides. Moreover, this study determined the chemical components of *A. longicuspis* extracts and antibiotic effect to fungal pathogen *C. gloeosporioides*.

## Results

**Figure 1:**
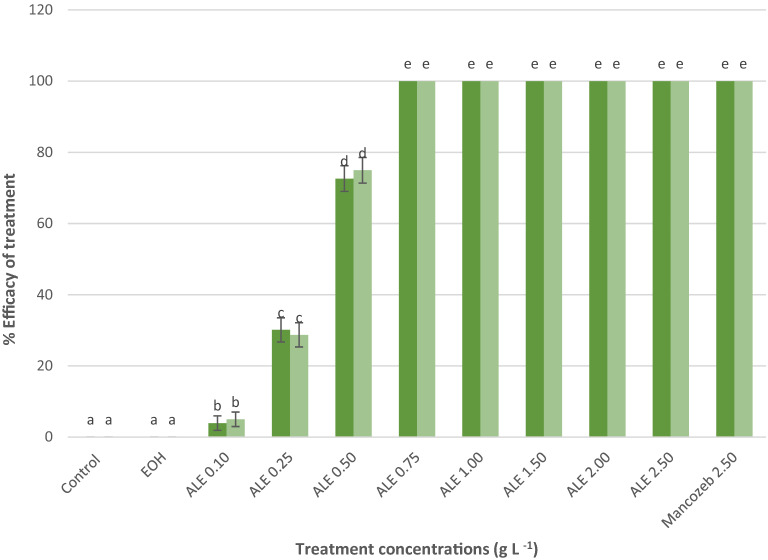
Percent inhibition on the mycelial growth of *C. gloeosporioides* 7 days after inoculation. Values followed with the same letter is not significantly different based on Tukey’s HSD test at p < 0.05. Green and light green represents trial 1 and 2. The vertical bars acted as standard error ( ±) of mean of the three biological replicates cited in the method section, *N* = 33 per trial.

**Figure 2:**
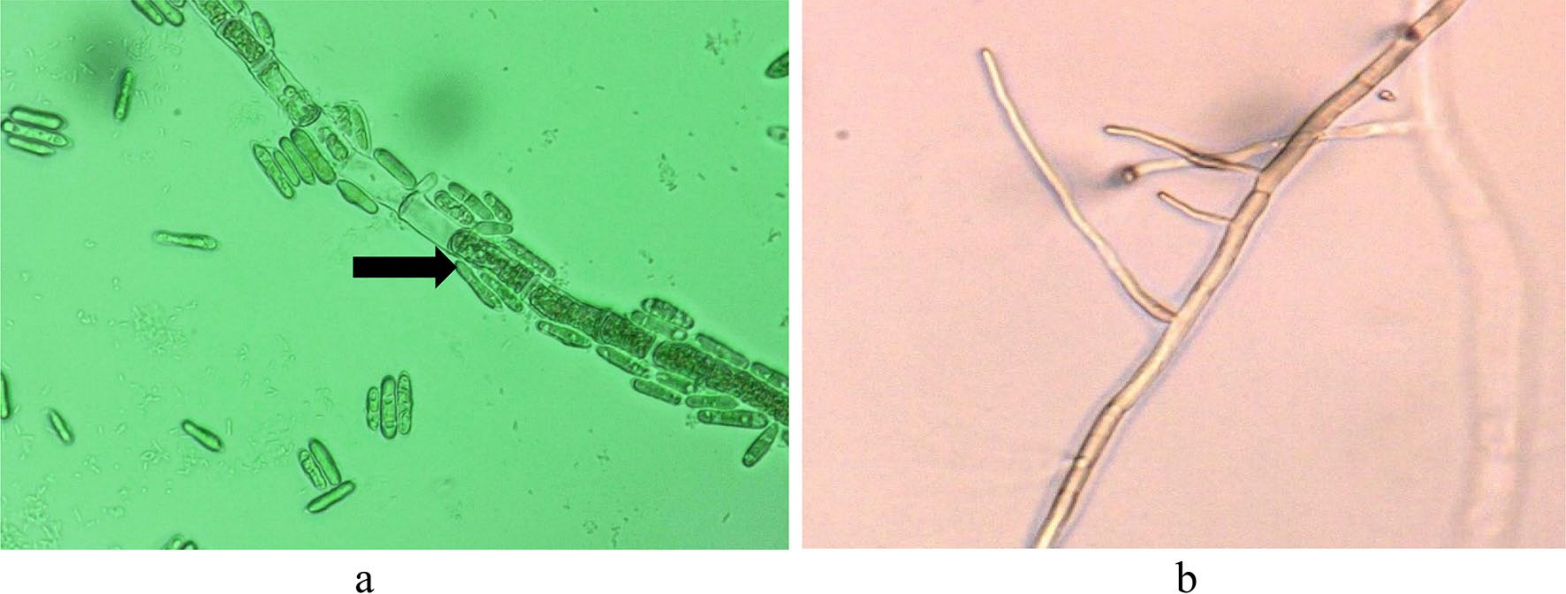
Mycelia of *C. gloeosporioides* **(a)** treated with ALE, **(b)** treated with water, 7 days after treatment as seen under the microscope.

In vitro analysis revealed that, ALE severely affected the mycelial growth of *C. gloeosporioides* with respect to the untreated control (Fig. 1). Complete inhibition of mycelial growth was observed starting from 0.75 g L^−1^ concentration of ALE and statistically comparable with the synthetic fungicides (Mancozeb) at 2.5 g L^−1^ concentration in terms of inhibitory effect on the mycelial growth. Lower concentration (0.75 g L^−1^) of ALE was needed in vitro to achieve complete inhibition of mycelial growth as compared with the conventional synthetic fungicide (Mancozeb 2.5 g L^−1^). The activity was directly organized and proportional, as the higher the treatment concentration, the greater the greater the effect on fungal inhibition, until it reaches the maximum rate of inhibition. Thus, adding more concentration of ALE will no longer affect the inhibition of the test fungi. The ALE-pathogen interaction showed the presence of mycelial blast, 7 days after treatment (Fig. 2a) as seen under microscope (Keyence, VHX-5000, USA) observations. However, *C. gloeosporioides* treated with water showed vigorous mycelial growth after treatment (Fig. 2b).

**Figure 3:**
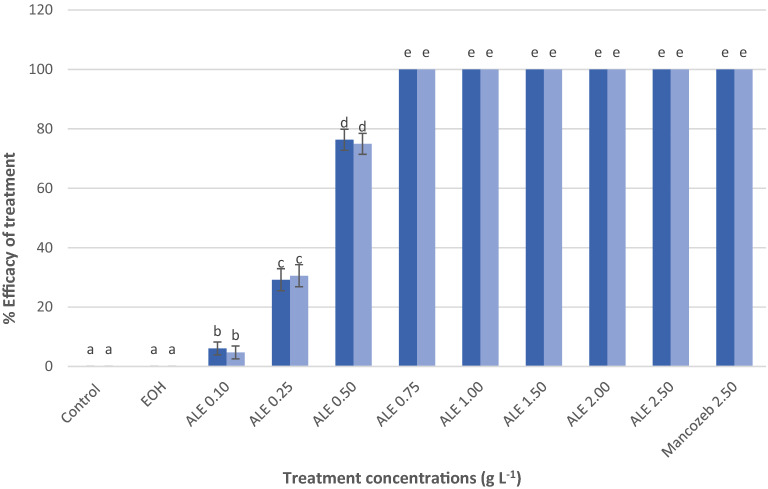
Percent conidial germination of *C. gloeosporioides*, 48 h after inoculation. Values followed with the same letter is not significantly different based on Tukey’s HSD test at p < 0.05. Blue and light blue bars represents trial 1 and 2. The vertical bars acted as standard error ( ±) of mean of the three biological replicates cited in the method section, *N* = 33 per trial.

**Figure 4:**
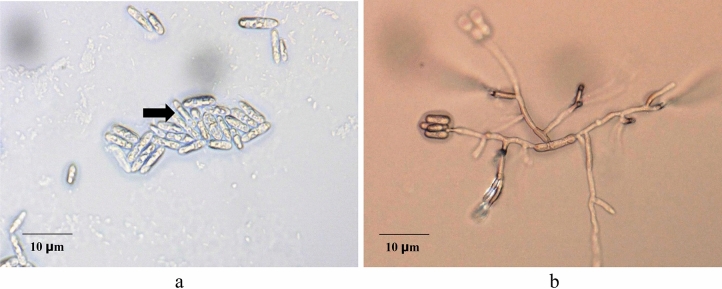
Conidia of *C. gloeosporioides* **(a)** treated with ALE **(b)** treated with water, 48 h after treatment as seen under the microscope.

The ALE at 0.75 g L^−1^ concentration completely inhibit the conidial germination of *C. gloeosporioides* (Fig. 3). Cytoplasmic discharge and conidial blast was vividly observed on *C. gloeosporioides* treated with ALE (Fig. 4a) but conidial germination was noticed at *C. gloeosporioides* treated with water (Fig. 4b), 48 h after treatment.

**Figure 5:**
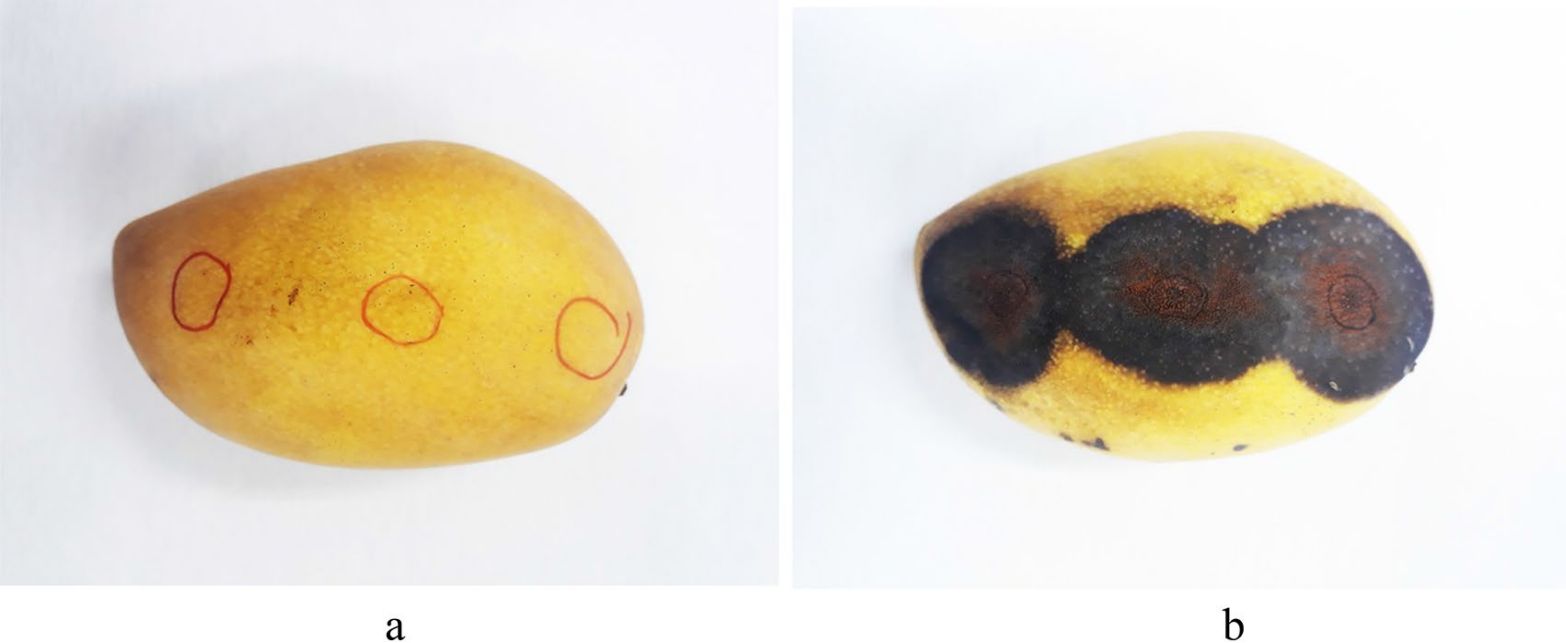
Mango treated with ALE **(a)** treated with *C. gloeosporioides* **(b)**, 7 days after treatment.

Meanwhile, after 7 days, mango fruit treated with ALE resulted no discoloration nor detectable phytotoxicity was observed even at highest concentration of 2.5 g L^−1^ (Fig. 5a). However, fruit treated with *C. gloeosporioides* suspension resulted in sunken, discolored, black lesions that looks like “alligator skin” on the surface of inoculation sites of mango (Fig. 5b).

**Figure 6:**
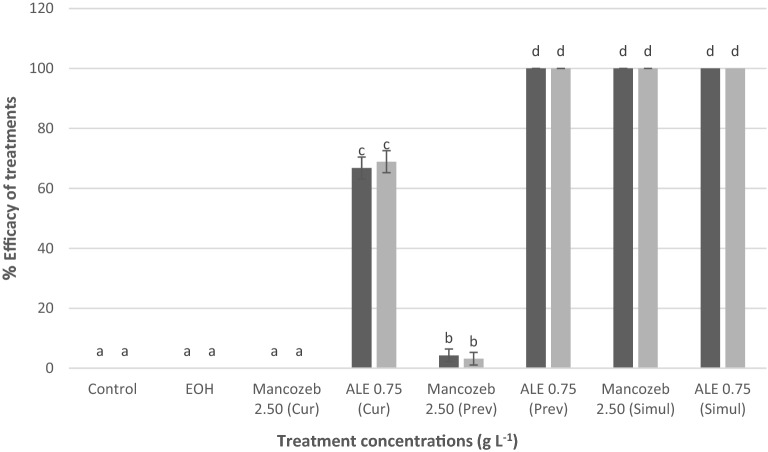
The efficacy of treatments 7 days after inoculation. Values followed with the same letter is not significantly different based on Tukey’s HSD test at p < 0.05. Black and gray bars represents trial 1 and 2. The vertical bars acted as standard error ( ±) of mean of the three biological replicates cited in the method section. *Cur* curative, *Simul* simultaneous, *Prev* preventative.

On in vivo test, direct application of ALE 24 h before, after and simultaneously with inoculation of *C. gloeosporioides* significantly reduced the anthracnose incidence on mango fruit (Fig. 6). The ALE gave greater efficacy against anthracnose of mango as compared to conventional synthetic fungicide. The curative effect of ALE at 0.75 g L^−1^ (66.81–68.89% efficacy) was far better than the synthetic fungicide (Mancozeb 2.5 g L^−1^)—having zero or no effect at all. Preventative activities of ALE at 0.75 g L^−1^ provided complete control against anthracnose of mango; synthetic fungicide only got 3.17–4.31% efficacy. However, ALE was statistically comparable with the synthetic fungicide in terms of efficacy and inhibitory effect (100% effective) in simultaneous activities. Generally, ALE as preventative, curative and simultaneous activities were relatively superior to commercially synthetic fungicide (Mancozeb) in terms of efficacy and potency.

Twenty chemical components were detected in ALE based on GC–MS analysis (Table 1). The six major components were the following: oleyl alcohol, methyl ether (42.04%), γ-sitosterol (15.85%), , 24-norursa-3.12-diene (5.62%), 1-octadecanol methyl ether (4.23%), *n*-pentadecanol (3.95%) and 2-vinyl-4h-1 3-dithiine (3.76%).

**Table 1:** Chemical composition of *Allium longicuspis* extracts.

RT	Name	% Area
5.173	6-Methylheptan-3-ol	3.73
5.277	3-Vinyl-1 2-dithiacyclohex-4-ene	0.64
5.444	2-Vinyl-4h-1 3-dithiine	3.76
6.048	Diallyl trisulfide	1.29
9.315	1-Methoxycarbonylethyl-5-methoxycarbonylpentyl ether	0.41
11.359	Tetradecanoic acid	0.56
13.669	*n*-Pentadecanol	3.95
15.200	*n*-Hexadecanoic acid	2.17
15.937	1-Chloro-4-(1-ethoxyethoxy)-2-methylbut-2-ene	0.96
17.104	9.12-Octadecadien-1-ol.(Z.Z)	1.29
17.269	Oleyl alcohol, methyl ether	42.04
17.764	1-Octadecanol methyl ether	4.23
17.919	Linoleic acid, methyl ester	1.25
26.077	Hexadecanoic acid 2-hydroxy-1-(hydroxymethyl)ethyl ester	1.75
28.746	Octadecanoic acid 2 3-dihydroxypropyl ester	0.88
32.374	Cholesta-4 6-dien-3-ol (3.beta.)-	1.38
32.767	1-Hexacosanol	2.84
32.975	Cholesterol	1.35
34.497	Campesterol	2.22
35.969	Γ-Sitosterol	15.85
36.656	9,19-Cyclo-9.beta.-lanostane-3.beta.25-diol	1.83
37.681	24-Norursa-3.12-diene	5.62

## Discussions

In the current investigation, ALE successfully inhibited growth of *C. gloeosporioides* (mycelia and conidia) in in vitro tests. Cytoplasmic discharge, mycelial and conidial blasts were clearly observed when applied with ALE. Parallel findings were observed by other scientists, which associates on the disruption and damaged of the fungal cell that leads into death^18–20^. The ALE at 0.75 g L^−1^ is hereby recommended, because at this concentration, complete fungal inhibition was observed with no detectable phytotoxicity on mango fruit. Although *Allium *spp. are known for its strong aroma, which is mainly caused by organosulfur compound. However, based on the phytotoxicity test conducted, the aroma of garlic can be detectable on the first 24 h after application, beyond that, no garlic aroma was observed. Further, those organosulfur compound was thermally unstable and naturally degraded by nature^21^.

Meanwhile, the minimum effective concentration (MEC) of ALE at 0.75 g L^−1^ concentration can be applied as protective, curative and simultaneous treatment on mango fruit to inhibit the anthracnose infection. The ALE exhibited a higher toxic effect on the test pathogen (*C. gloeosporioides*) even at a low volume concentration (0.75 g L^−1^) compared with synthetic fungicide (Mancozeb 2.5 g L^−1^) as a standard control. In fact, current results showed that ALE is thrice lower than the synthetic fungicide (Mancozeb) in terms of volume concentration to achieved complete control in simultaneous and preventative application. This implies that the major components and its derivatives may have interacted in a synergistic manner; accelerating its toxic effect that leads into death. The current results and the previous findings of other scientist support the potential utilization of *Allium *spp. (wild and traditional type) extracts against fungi and other microbial pathogens^22–24^.

The inconsistency on the results and/or effect of treatments (ALE and Mancozeb) used in in vitro and in vivo were explained by the structural level of complexity and absorptive capacity of two different media [agar (in vitro) and mango fruit (in vivo)]. The waxes (epicuticular wax layer), cuticular plates, microdomains (crystal and amorphous layer) and other physiological characteristic presents in mango peel was acted in synergistic manner; causing a low to acute toxicity of all the treatments (ALE and Mancozeb) used against the test pathogen^25–28^. There is also a strong evidence that *C. gloeosporioides* already developed resistance to Mancozeb, based on curative and preventative results. The same results were observed by Spalding et al*.*^29^, Kumar et al*.*^30^, Brent et al*.*^31^ which links the resistance of fungal pathogens of mango to conventional synthetic fungicides, including Mancozeb. On the other hand, higher efficacy of treatments (ALE and Mancozeb) was noticed in preventative over the curative test result. This could be due to the fact that, the fungal pathogen in curative tests has already established or in-set infection prior to the application of treatments. To quote “prevention is better than cure”.

On the other hand, one out of six major components of the identified compound from ALE was known for its biological activities. First, gamma sitosterol (steroid) was known for its anti diabetic activities^32^ and known potent inhibitor of complement C1 component^33^. It has been reported for anticancer activity through growth inhibition of the cancer cells. It also has cytotoxic effect against colon and liver cancer cell lines thru regulation and induction of the apoptotic pathways of the cell. Second, 2-vinyl-4h-1,3-dithiine, a member of dithiins compound^34^. Dithiins are organosulfur compound formed thru the breakdown of allicin from a garlic^35^. The toxic activities of garlic extract may be rationalized by presence of organosulfur and its derivatives^36^. However, oleyl alcohol methyl ether, 24-nourse-3,12-diene and 1-octadecanol methyl ether is a new compound whose biological activity is yet to ascertained.

Plant extracts as fungicide present two major characters. First, safe, natural and eco-friendly; second was less resistance development against target organisms^37^. In general, an organism can able to developed resistance into pure chemical compound but not in to a complex or mixture compounds^37–38^. Complex or mixture of compounds will further strengthen its effect on the target organisms. Such complexities were favorable in the broad target spectrum, because different organisms respond differently into single compounds^39^. Further, it reduces the potential development of genetic resistance and/or development of behavioral desensitization of an organism^40^.

*A. longicuspis* possessed excellent antifungal effects against plant pathogenic fungi (*C. gloeosporioides*) and can be used as a replacement and/or auxiliary with conventional synthetic fungicide in the market. Their synergistic effect in combined with other plant extracts however, should also be investigated for future research. Other factors such as mode of action and formulating fungicide derived from plant based products in such a way that improves the product stability and potency. This work is timely and relevant as an alternative cure and control the deadest pathogen, facing the world today.

## Methods

### Garlic and extracts preparation

The *A. longicuspis* were collected in Ilocos Sur, Philippines 17° 20′ N  120° 35′ E. It has a white to purple scales, bulbs are ovoid with a diameter of 2–2.5 cm consisting of compactly crowded truncated and angular tubers. Fresh *A. longicuspis* were extracted using ethanol (EOH) as a solvent, following the method of Mangoba and Alvindia^17^ with modifications. The *A. longicuspis* extracts (ALE) were vacuum filtered and evaporated using rotary evaporator. Thereafter, the obtained extracts were stored at − 80 °C until gas chromatography and mass spectrometry (GCMS) analysis were performed.

### Chemical profiling

Chemical profiling of ALE were performed following the method described by Ji et al*.*^41^ with modifications, using GC/MS by Shimadzu. Gas chromatography was preformed using Shimadzu GC2010 model together with a Shimadzu QP2010 for mass spectrometer. Individual constituents of the ALE were characterized based on the retention time (RT) and fragmentation pattern of the mass spectra in the system of National Institute of Standards and Technology (NIST) libraries. The percent composition of the compounds were calculated from peak areas of gas chromatography.

### Isolation of fungal pathogen

The ripe mango cv. “Carabao” fruit showing diseases incidence (anthracnose lesions) were acquired from local market in Nueva Ecija 15° 35′ N  121° 00′ E, Philippines. The anthracnose lesions of 1 cm^2^ were directly removed from the infected ripe mango fruit using sterile scalpel. The infected mango tissues were dipped in 30% sodium hypochlorite for 1 min, rinsed thrice in sterile water for one minute and placed on sterile filter paper. Infected mango tissues were directly plated in potato dextrose agar (PDA) and placed in convection incubator (Binder: DB-56, Germany) for seven days at 25 ± 2 ℃ and 70 ± 5% relative humidity.

### In vitro tests

The efficacy of ALE on the mycelial growth of the fungal isolate was evaluated following the method described by Chen et al*.*^42^ with modifications. The PDA was mixed with ALE at a ranged of 0.1 to 2.5 g L^−1^ concentrations. The concentration was prepared based on the procedure by Ali et al.^43–44^. PDA plates treated with SW were served as negative control 1 and EOH for negative control 2. Ethanol as a solvent was used to determine whether it has adverse effect on the test pathogen and fruit or not. However, PDA treated with synthetic fungicide (Mancozeb at 2.5 g L^−1^) was used as a positive control.

A ten day-old *C. gloeosporioides* mycelial disc of (5 mm) was transferred into PDA plates (treated and control) and incubated as described above. The mycelial growth of *C. gloeosporioides* was measured 7 days after treatment by measuring colony diameter using a digital caliper. The efficacy of the treatment (ET) on mycelial growth was determined using the formula ET = (CdNC1 – CdT)/ CdNC1 × 100; whereas: CdNC1 = is the colony diameter of the negative control 1 and CdT = colony diameter of treated with ALE and synthetic fungicide ^5^. Two trials were arranged using complete randomized design (CRD) with three replications per treatments.

Conidial germination was assessed following the procedure described by Alvindia et al.^5–6^ with some modification. Test pathogen (*C. gloeosporioides*) was cultured on PDA for ten days and incubated as described above. After 10 days, the *C. gloeosporioides* plates was flooded with 5 ml of SW and was softly scraped with sterile glass L-rod. The fungal suspension was filtered using four layer of sterile cheese cloth to separate the conidia from PDA. The conidial concentration was calculated and adjusted using hemocytometer of 10^7^ conidias L^−1^. A 100 μl of the conidial suspension of the fungal pathogen was spread on water agar treated with 0.1–2.5 g L^−1^ concentration of ALE. Conidia receiving with SW was used as negative control and EOH for negative control 2 while conidia treated with synthetic fungicide (Mancozeb) was served as the positive control. Under the light microscope, 100 conidia were counted after 48 h. Percent conidial germination was calculated by dividing the number of germinated conidia over the total conidia counted and multiplied by 100^5–6^. Subsequent abnormal bulb-like formations, bursting and spore swelling were recorded. The bio-assays were arranged as described above with two trials.

### Phytotoxicity of ALE and pathogenicity of *C. gloeosporioides*

The possible toxicity of ALE to mango fruit was evaluated. The 20 μl of ALE at various concentration (0.75–2.5 g L^−1^) was applied on different inoculation sites (top, middle and bottom) of mango fruit. On the other hand, the isolated fungal pathogen (*C. gloeosporioides*) was tested artificially on mango fruit for the assessment of pathogenicity tests^5–6^. Untreated mango fruit was served as control. Treated fruit were placed in a sterile container and kept for seven days at 25 ± 2 ℃ and 70 ± 5% relative humidity. After seven days, fruit were assessed for damage characteristic of pathogenicity and phytotoxicity test. The test was replicated thrice with two trials.

### Postharvest application

The efficacy of ALE on mango fruit were evaluated using the method describe by Chen et al*.*^42^ with modifications. Ripe mango fruit cv. Carabao were rinsed twice in SW and air dried at 25 ± 2 ℃ and 70 ± 5% RH, to remove other contaminants that might affect the quality of the fruit. Three mango fruit were used with three replications per treatment. Three inoculation sites of each mango fruit were determined (top, middle and bottom). Each inoculation sites were marked and lightly wounded using sterile “insect pins” (No. 1). The sterile insect pins were pricked once on mango fruit skin, creating a slight wound of ≤ 1.50 mm diameter. Thereafter, 10 μl of the pathogen isolate [7 day old *C. gl oeosporioides* suspension (10^7^conidia/mL)] and treatments (Mancozeb at 2.5 g L^−1^ and ALE 0.75 g L^−1^) were applied either 24 h before (preventative test), simultaneously and after 24 h (curative test) in each inoculating site. Fruit only inoculated with *C. gloeosporioides* suspension was served as control 1 while EOH was served as control 2. Treated fruit were kept in sterile plastic box (25 × 40 cm) at 25 ± 2 ℃ and 70 ± 5% RH. The effect of ALE on *C. gloeosporioides* was assessed 7 days after treatment, by measuring lesion diameter on the fruit surface using a digital caliper. The efficacy of the treatments (ET) was evaluated as described above. The bioassays were prescribed as described above with two trials.

### Statistical analysis

Data gathered from the experiments were statistically compared and analyzed using Tukey’s HSD test, applying analysis of variance (ANOVA) at P < 0.05 significant differences. It was carried out using SPSS software (IBM 20 for IOS, NY, USA).
