# Publisher Correction: Bioactivities of *Allium longicuspis* Regel against anthracnose of mango caused by *Colletotrichum gloeosporioides* (Penz.)

**DOI:** 10.1038/s41598-020-78515-8

**Published:** 2020-12-15

**Authors:** Dionisio de Guzman Alvindia, Mark Anthony Angeles Mangoba

**Affiliations:** 1 Food Protection Division, Philippine Center for Postharvest Development and Mechanization, Department of Agriculture, Muñoz, Nueva Ecija Philippines; 2 Department of Bio‑Resource and Food Science, College of Life and Environmental Sciences, Konkuk University, Seoul, South Korea; 3 Center for Natural Sciences and Environmental Research (CENSER), De La Salle University, Taft Ave., Manila, Philippines

Correction to: *Scientific Reports* https://doi.org/10.1038/s41598-020-68399-z, published online 09 July 2020

The original version of this Article contained a typographical error in the spelling of the author Dionisio de Guzman ALVINDIA, which was incorrectly given as Dionisio Guzman de ALVINDIA. The email address should be ma_mangoba@yahoo.com instead of ma_mangoba@yhaoo.com. This has now been corrected in the PDF and HTML versions of the Article.

